# One-Pot Preparation of Easily Dispersible Hexagonal Mg(OH)_2_ Modified with THPS and Its Flame-Retardant EVA Copolymer

**DOI:** 10.3390/ma18214847

**Published:** 2025-10-23

**Authors:** Xia Liu, Haihui Xu, Jinyang Chen

**Affiliations:** School of Environmental and Chemical Engineering, Shanghai University, 99 Shangda Road, Shanghai 200444, China; liuxiaqaq@shu.edu.cn (X.L.); mzshxhh@shu.edu.cn (H.X.)

**Keywords:** magnesium hydroxide, modification, THPS, flame retardant, thermal properties

## Abstract

As an eco-friendly flame-retardant additive, magnesium hydroxide (MH) is widely employed in low-smoking, halogen-free polymer materials due to its environmentally benign nature. In order to enhance flame retardancy performance, the modified MH was modified with tetrakis(hydroxymethyl)phosphonium sulfate (THPS) by a one-pot hydrothermal method. The resulting morphology was characterized using scanning electron microscopy (SEM), and it shows the dispersion of nanometer particles and almost no aggregation. The X-ray photoelectron spectroscopy (XPS) along with Raman spectroscopy show that the THPS is connected with the Mg(OH)_2_ by chemical bond. The sample was incorporated into ethylene–vinyl acetate (EVA) to evaluate the flame retardancy was assessed via limiting oxygen index (LOI) and vertical burning tests (UL-94). The results show that THPS modified MH effectively enhanced the flame retardancy, achieving a V-0 rating and an LOI value of 31.3%. In addition, the composites retain good mechanical integrity. The thermal analysis with TGA and DTG shows the formation of the MgO decomposition product, along with water vapor and phosphorus-containing radicals released by modified MH in the combustion process, forming a strong flame-retardant protective layer. In addition, the maximum smoke density of EVA/MHP-3 composite was 155.4, lower than 411.3 for EVA/MH, with a 62.2% reduction in total smoke production. The result shows that THPS is effective for improving the flame-retardant efficiency of inorganic metal hydroxide in polymer composites.

## 1. Introduction

Ethylene–vinyl acetate (EVA), a versatile copolymer, is widely used in coatings, electronic components, pharmaceuticals, and packaging materials due to its favorable mechanical strength, corrosion resistance, adhesive properties, and low dielectric constant [1,2,3]. However, as an organic polymer, EVA shows inherent flammability and combustibility [4]. During combustion, it generates a large amount of toxic gases and dense smoke, posing significant risks to both environmental safety and human well-being [5]. Accordingly, strengthening the fire-retardant properties of EVA is essential to improve its safety and broaden its applicability in demanding environments.

In recent years, various types of flame-retardants have been developed, including phosphorus-based flame-retardants (red phosphorus [6], hypophosphite [7], phosphaphenanthrene [8,9], phosphorus-containing imidazolium compounds [10], pyrophosphate [11]), halogenated flame-retardants (BFRs [12,13,14], CFRs [15]), nitrogen-containing flame-retardants [16,17], inorganic flame-retardants [18,19,20], and organic flame-retardants [21,22]. Among them, inorganic nanoparticles offer advantages such as plentiful raw materials, low cost, and straightforward synthesis. Among them, magnesium hydroxide (MH) has found widespread application as a green inorganic flame-retardant owing to its plentiful resources, environmental friendliness, low toxicity, non-corrosiveness, acid-free, and cost-effectiveness in organic polymer materials. Meanwhile, as a halogen-free flame-retardant, MH offers a higher decomposition temperature (340 °C) [23,24,25] and the ability to release water vapor during thermal decomposition. The released water vapor reduces surface temperature and dilutes flammable gases [26]. Moreover, the in situ formation of the magnesium oxide (MgO) during thermal decomposition provides a physical protective barrier, hinders the diffusion of flammable gases, and prevents pyrolysis of the polymer matrix. Nevertheless, as an inorganic filler, MH presents certain disadvantages, such as poor compatibility and dispersion in polymers, which will result in the deterioration of the mechanical properties of polymer composites [27,28]. Additionally, the flame-retardance of pure MH is primarily based on the endothermic expansion and water vapor release, resulting in weak flame-retardant performance [29]. Therefore, it is essential not only to improve the flame-retardant capability of MH but also to enhance the mechanical performance of polymers.

Phosphorus-containing compounds can be combined with MH flame-retardants to achieve synergistic effects, enhancing flame-retardant performance [30,31] through gas-phase and condensed-phase flame-retardant mechanisms [32,33,34]. Phosphorus-containing species released during combustion can react with gas-phase radicals (·OH, ·H, etc.), effectively terminating the flame propagation process. In the condensed phase, the stable phosphorus-carbon char layer forms and acts as a barrier layer to block heat and pyrolysis products. Furthermore, the phosphorus compounds promote the polymer matrix’s carbonization, improving the thermal stability and structural integrity of the residual char. The dual mechanism improves flame retardancy by slowing down the pyrolysis process as well as shielding the substrate beneath from additional deterioration. To date, various methods have been developed to obtain phosphorus-modified magnesium hydroxide [35,36,37]. Zhao et al. [38] studied a hierarchical core–shell architecture flame-retardant, in which magnesium hydroxide is modified by cyclic cross-linked polyphosphazene (MH@CPZN), with the samples significantly reinforcing the flame resistance of styrene-butadiene-styrene asphalt (SBSA) while also elevating the asphalt’s fire safety and smoke suppression performance. Yao et al. [39] reported that MH modified with PZN (MH-PZPI-BA) showed enhanced flame-retardant performance and improved smoke suppression properties. Among various phosphorus-based compounds, Tetrakis(hydroxymethyl)phosphonium sulfate (THPS) is a colorless and transparent liquid that readily decomposes into non-toxic substances, making it an eco-friendly and sustainable compound [40,41]. THPS contains four reactive hydroxymethyl groups and is capable of inserting itself into the interlayer spaces of MH to increase the generation of a stable protective layer. Meanwhile, as a halogen-free phosphorus source, THPS can further enhance the flame-retardant effect of magnesium hydroxide through gas-phase free radical capture and solid-phase char formation [42].

A one-pot method was applied to prepare highly dispersed, hexagonal plate-like magnesium hydroxide modified with THPS. The as-prepared MH exhibits superior dispersion in polymer matrices, which can maintain mechanical performance and improve interfacial compatibility. The application of THPS in flame-retardant modification is rare, with most previous studies focusing on industrial water treatment. Introducing THPS for MH modification, therefore, represents a novel strategy that not only enhances flame-retardancy and smoke suppression but also provides an eco-friendly and practical route for the development of halogen-free flame-retardant polymer composites, offering insights for further applications in other polymer systems.

## 2. Materials and Methods

### 2.1. Materials

Every chemical employed in the experiments was of analytical quality and utilized as received, without additional purification. The magnesium chloride (MgCl_2_, 97%) was supplied by Shanghai Dibo Biotechnology Co., Ltd. (Shanghai, China). Sodium hydroxide (NaOH, AP), Stearic acid, Aqueous ammonia (28%), and THPS were obtained from Shanghai Titan Technology Co., Ltd., Shanghai, China. The Ethylene–vinyl acetate copolymer (V6110M) was obtained from Yangzi Petrochemical Co., Ltd. (Nanjing, China). The water used throughout the experiments was prepared in the laboratory as deionized (D.I.) water.

### 2.2. Synthesis of MH

Magnesium hydroxide was prepared with a double-dripping mode [43,44]. First, the 100 mL 3 mol/L MgCl_2_ aqueous solution was mixed with the NH_3_⋅H_2_O (28%) in a three-necked flask by the double-dripping mode with a mole ratio of the Mg^2+^: OH^−^ of 1:2.4. After dripping, stirring of the mixture was carried out for 1 to 2 h at 20 °C. Then, the mixture was passed through a filter and washed to obtain the wet filter cake of magnesium hydroxide.

### 2.3. Preparation of Modified Mg(OH)_2_

**Figure 1:**
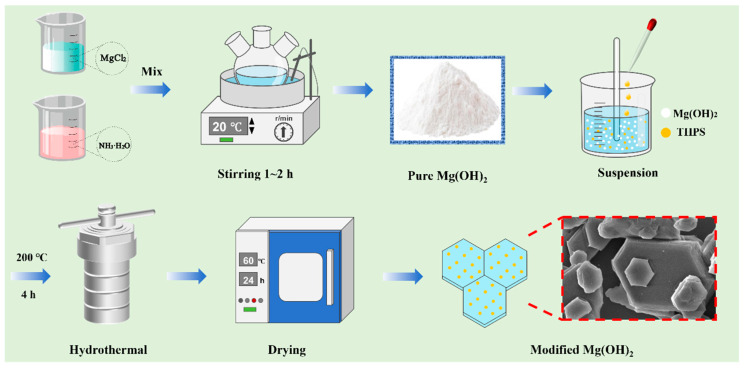
The preparation process of THPS modified the Mg(OH)_2_.

Modified magnesium hydroxide was prepared via a one-pot method at high temperature. First, the MH wet filter cake obtained from step 2.2 was dispersed in 100 mL of a 5 M NaOH solution to form a suspension. After stirring at 1800 rpm for 30 min to ensure uniform dispersion, THPS was subsequently added as a modifier. The suspension was then transferred to a Teflon-lined stainless-steel autoclave (Xingjian Chemical Machinery Co., Ltd., Taizhou, China) at 200 °C for 4 h. After naturally cooling to ambient temperature, the product underwent filtration, was washed thoroughly, and then dried at 60 °C over 24 h to obtain highly dispersible modified Mg(OH)_2_. The modified products were labeled as MHP-X, where “X” represents the weight percentage of THPS added relative to the mass of the Mg(OH)_2_. For example, MHP-1, MHP-3, and MHP-5 contain 1 wt.%, 3 wt.%, and 5 wt.% of THPS. The preparation process is shown in Figure 1.

### 2.4. Preparation of EVA Composites

Melt blending of EVA with 60 wt.% modified or unmodified Mg(OH)_2_ was performed on a two-roll mill (KY-3203D-160, Dongguan Kaiyan Machinery Technology Co., Ltd., Dongguan, China) to produce the composites. The compounding was conducted at 145 °C for 15 min with a nip gap of 1 mm and a roller speed of 20 rpm to ensure uniform dispersion of the fillers. The blended materials were then hot-pressed into sheets at a temperature of 170 °C and a pressure of 10 MPa for five minutes with a plate vulcanizing press, followed by cooling to ambient temperature under pressure.

### 2.5. Characterization

The XRD was obtained through using a Bruker D8 ADVANCE diffractometer equipped with Cu Kα radiation, operated at 40 mA and 45 kV (Rheinstetten, Germany). The scanning rate was 8°/min, with a 2θ range of 10 to 90°. The micromorphology was observed by scanning electron microscopy (SEM, German ZEISS Sigma 300, Oberkochen, Germany), with a 3 kV accelerating voltage, and a Au coating onto the sample was completed before testing. Elemental composition was determined by energy-dispersive spectroscopy (EDS) at 15 kV.

Raman spectroscopy (HORIBA, Kyoto, Japan) was successfully obtained with a Horiba Scientific LabRAM HR Evolution and a 514 nm wavelength excitation light. X-ray photoelectron spectroscopy (XPS) measurements were conducted on a Thermo Scientific spectrometer (Thermo Fisher Scientific, MA, USA) with K-alpha radiation.

Thermal stability was investigated using a HITACHI STA200 simultaneous thermal analyzer,(Hitachi, Tokyo, Japan) combining thermogravimetric analysis (TGA), with samples gradually heated starting from an ambient temperature and continuing up to 800 °C at a rate of 10 °C per minute.

Using a TTech-GBT2408 oxygen index apparatus (TESTech Instrument (Suzhou) Technologies Co., Ltd., Suzhou, China) and following the Chinese standard GB/T 2406.2-2009 [45], the limiting oxygen index (LOI) of the sample was determined. The sample size was 150 mm × 7 mm × 3 mm. Vertical combustion (UL-94) measurements were conducted according to GB/T 2408-2021 [46] using a TTech-GBT2406-2T instrument (TESTech Instrument (Suzhou) Technologies Co., Ltd., Suzhou, China), with a specimen size of 125 mm × 13 mm × 3 mm.

Smoke density measurements were performed using a TTech-GBT8323-2 smoke density test instrument (TESTech Instrument (Suzhou) Technologies Co., Ltd., Suzhou, China) following ASTM E662 [47] under no-flame conditions. Specimens (75 × 75 × 3 mm^3^) were positioned horizontally and subjected to an external heat flux of 25 kW·m^−2^.

An MT-4000D1 universal testing machine (Jiangsu Mingtuo Testing Machinery Co., Ltd., Jiangsu, China) was employed to evaluate the tensile behavior of the materials, following the ASTM D638 standard [48], with a minimum of five independent tests performed for each sample (115 mm × 6 mm × 1 mm).

## 3. Results and Discussion

### 3.1. Optimal Modified Conditions for MH

#### 3.1.1. Morphology

**Figure 2:**
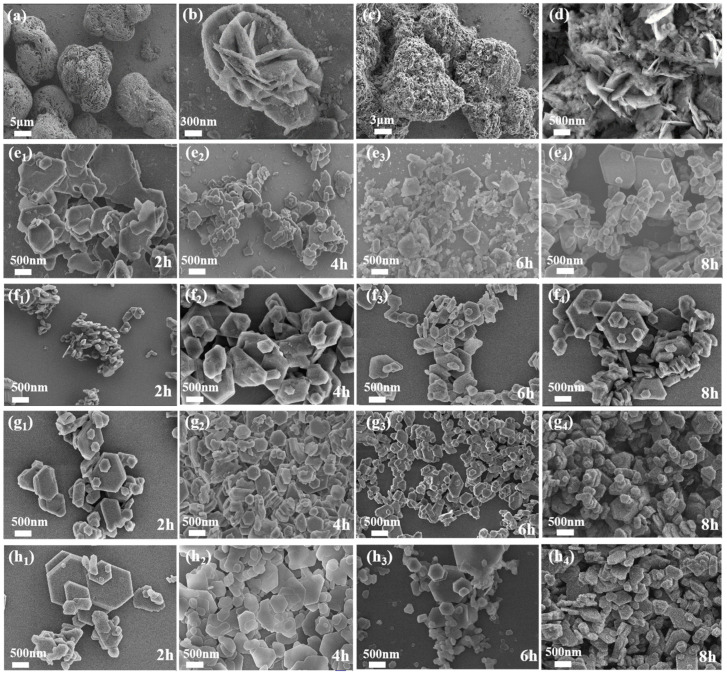
SEM images of magnesium hydroxide prepared under different conditions: (**a**,**b**) unmodified sample at 20 °C; (**c**,**d**) sample modified at low-temperature (60 °C) and 8 h; (**e**–**h**) samples modified under hydrothermal conditions at; (**e_1_**–**e_4_**) 160 °C; (**f_1_**–**f_4_**) 180 °C; (**g_1_**–**g_4_**) 200 °C; and (**h_1_**–**h_4_**) 220 °C.

Magnesium hydroxide was initially prepared at 20 °C without any modifier. As shown in the SEM images (Figure 2a,b), the sample exhibited severe agglomeration and disordered stacking of lamellar particles. Further modifications were conducted at different temperatures to improve particle morphology and dispersion. A low-temperature reaction (60 °C) and a high-temperature hydrothermal method were studied for comparison.

At 60 °C for 8 h, the product showed large, irregular flakes with extensive stacking and aggregation (Figure 2c,d). Although the lamellar structure was retained, the limited diffusion of THPS restricted the interaction with the magnesium hydroxide surface, resulting in poor modification efficiency and the formation of larger clusters. The results indicate that low-temperature modification fails to effectively improve particle morphology.

High-temperature conditions lead to a decrease in the viscosity of the solution and an increase in the ion diffusion rate. As a result, the growth rate of crystals surpasses the nucleation rate. This means that larger particles grow while small particles dissolve. Consequently, the process obtains regular hexagonal, sheet-like structures of magnesium hydroxide [28,49]. Meanwhile, high temperatures facilitated THPS diffusion, increasing the contact with MH and reducing surface energy. Therefore, the high-temperature hydrothermal method produced modified magnesium hydroxide with more uniform shapes, larger particle sizes, and higher crystallinity, making it more favorable than the low-temperature process.

It is known that increasing the temperature or prolonging the hydrothermal treatment time can enhance the hexagonal plate-like morphology of magnesium hydroxide and improve particle dispersion [50]. The appropriate temperature and time to control the morphology and dispersion of modified magnesium hydroxide particles should be determined.

As shown in Figure 2, the SEM observations reveal the morphology of magnesium hydroxide modified under varying hydrothermal temperatures and treatment times. The modified magnesium hydroxide particles prepared at 160 °C (Figure 2(e_1_–e_4_)) showed uneven sizes, irregular flakiness, and agglomeration. The hexagonal lamellar structure started to appear after 8 h.

Increasing the temperature to 180 °C (Figure 2(f_1_–f_4_)) improved particle dispersion. After 4 h, a distinct thickness of the lamellar structure was observed, but the particles were small, making the lamellae susceptible to stacking. After extending the time to 8 h, the size of the lamellar crystals tended to be uniform.

Under the hydrothermal temperature at 200 °C (Figure 2(g_1_–g_4_)), a clear lamellar structure can be observed in 2 h, and the dispersibility is obviously improved. With prolonged exposure to 4 h, the growth orientation of modified magnesium hydroxide particles became more uniform, giving rise to distinct morphological features, a tight particle size range, and greatly enhanced dispersion. Prolonging the hydrothermal time maintained the structural integrity without evident aggregation or morphological degradation. However, when the temperature was raised to 220 °C (Figure 2(h_1_–h_4_)), signs of melting appeared at the edges of the particles, and the corners became rounded. This indicates that too high a temperature may lead to structural degradation. Therefore, based on morphology and dispersion, 200 °C for 4 h was determined to be the optimal hydrothermal treatment condition.

#### 3.1.2. Crystal Structure

**Figure 3:**
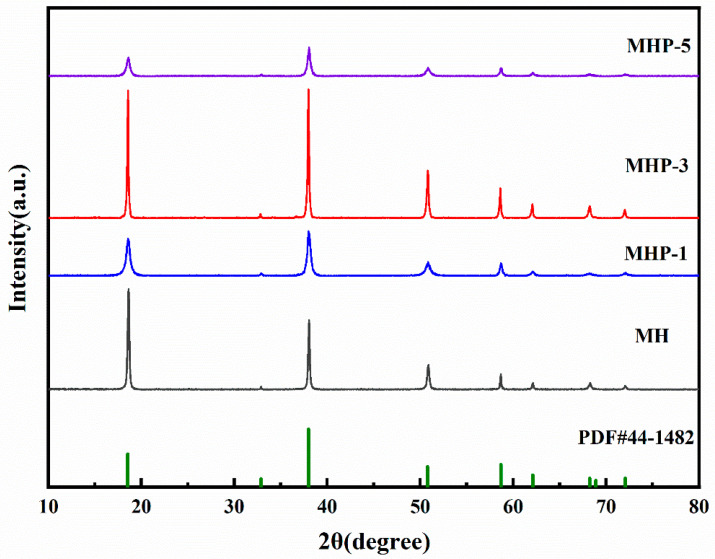
The XRD patterns of MH, MHP-1, MHP-3, MHP-5, and the standard MH.

The XRD patterns of Figure 3 show pure MH, MHP-1, MHP-3, and MHP-5 to study the crystal structure of different THPS addition contents. All diffraction peaks can be indexed to the standard XRD pattern of MH (PDF#44-1482), confirming the formation of a hexagonal lamellar crystal structure. The average crystallite size (*D*) was calculated using the Debye–Scherrer [51] equation, as shown in Equation (1):

(1) $$D=\frac{K\lambda }{\beta \cos\theta }$$

where the Scherrer constant (*K*) is 0.89, the Cu Kα X-ray wavelength (*λ*) is 1.5405 Å, *β* represents the peak broadening at half maximum (FWHM), and *θ* is the Bragg diffraction angle. With the calculation, 2*θ* values of 18.6 and 38° were obtained for specific diffraction peaks. The intensity ratio I_001_/I_101_ extracted from the XRD patterns indicates the preferred orientation of the crystal planes. The crystal parameters and grain size of MH modified with varying amounts of THPS were obtained from XRD patterns. The results gathered from the analysis are conveniently displayed in Table 1.

The growth rates of different crystal planes vary during the crystallization process, especially for the characteristic diffraction peak (001) plane in the hexagonal MH structure, which is known for its high surface energy [52,53]. According to Table 1, the sample without hydrothermal treatment exhibited a relatively disordered crystal structure, with an I_(001)_/I_(101)_ ratio of 0.71. The crystallites were still in the initial growth stage, with the measured particle diameter (D50) averaging 1.477 μm. After hydrothermal treatment at 200 °C, the I_(001)_/I_(101)_ ratio increased to 1.71, indicating improved crystal orientation and growth.

**Figure 4:**
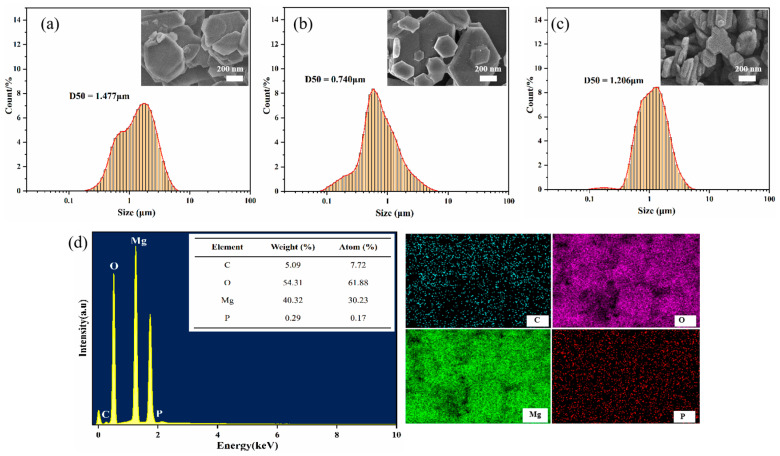
The images of the products prepared with different doses of THPS at 200 °C: (**a**) MHP-1, (**b**) MHP-3, (**c**) MHP-5, (**d**) EDS spectra and elemental mapping of MHP-3 at 4 h.

Figure 4 gives the corresponding EDS spectrum and elemental mappings. In the MHP-1 sample, all diffraction peaks in the XRD pattern became weaker and broader, and the size of the crystallites calculated reached 16.15 nm. The SEM image in Figure 4a shows irregular plate-like structures with broken edges, where the particle size averaged (D50) is 1.477 μm, suggesting inhibited crystal growth and the presence of structural defects. With the THPS concentration increased to 3 wt.% (Figure 4b), all diffraction peaks of MHP-3 became stronger and narrower, and the I_(001)_/I_(101)_ ratio was 0.99. Moreover, the crystallite size increased to 45.26 nm, while the average particle size decreased to an optimal of 0.740 μm. The SEM images show a uniform and well-defined hexagonal plate-like structure, indicating optimal crystal orientation and growth. The result can be attributed to the preferential adsorption of THPS on the high-energy (001) surface, potentially restricting crystal growth along this direction, resulting in a more stable structure due to a decrease in polarity [54].

However, increasing the THPS content to 5 wt.% (Figure 4c), the particles became smaller and showed a tendency to aggregate. Meanwhile, the XRD peaks weakened and broadened again, the crystallite size decreased to 17.42 nm, and the particle size increased to 1.206 μm, indicating a more disordered structure with increased defects. The result may be ascribed to the too much adsorption of THPS, which inhibited crystal growth and reduced crystallinity.

Therefore, the THPS concentration of 3 wt.% effectively enhanced the crystallinity and orientation of the MH, promoting the formation of an ordered structure and minimizing defects.

### 3.2. Modification Reaction Mechanism

**Figure 5:**
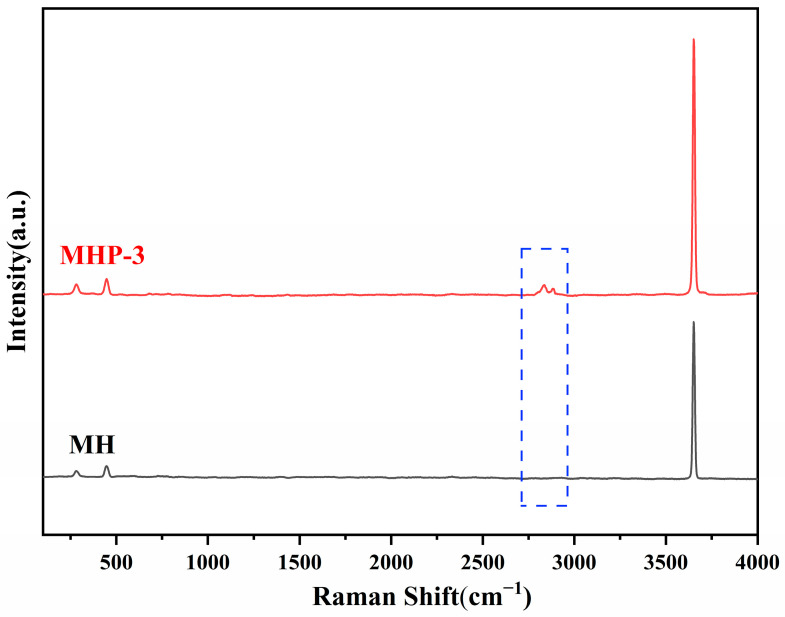
Raman spectra of MH and MHP-3 samples.

Figure 5 shows the Raman spectra of pure MH and MHP-3. The symmetric stretching mode corresponding to –OH is appearing at 3652 cm^−1^. The peaks observed at 282 and 447 cm^−1^ correspond to the vibrational modes of MH [55,56]. A new peak observed (2867 cm^−1^) in the MHP-3 within the range of 2800–3000 cm^−1^, is assigned to the C–H stretching vibrations [57,58,59,60].

**Figure 6:**
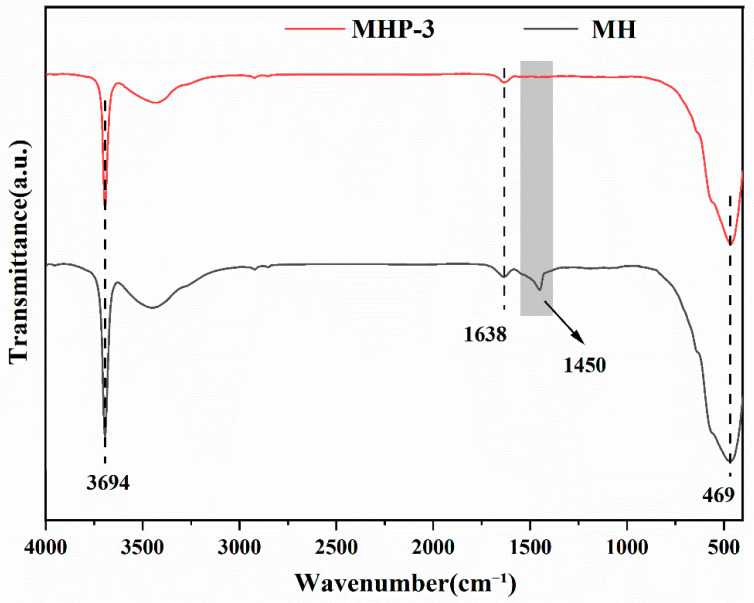
FTIR spectra of MH and MHP-3 samples.

FTIR offers valuable information on functional groups and bonding changes, enabling the analysis of chemical interactions between magnesium hydroxide and THPS. Figure 6 shows the FTIR (for MH and MHP-3) spectra. The remarkably sharp and intense peak at 3694 cm^−1^ is assigned to the O–H stretching vibration of MH, while the peak at 469 cm^−1^ can be assigned to the Mg–O vibration [61]. Additionally, a weak peak at 1638 cm^−1^ can be assigned to the bending vibration of crystal water (H–O) and P–C stretching vibration. It is important to point out clearly that the peak at 1450 cm^−1^ can be ascribed to the –OH bending vibration of magnesium hydroxide, which disappeared in MHP-3, indicating that the –OH groups of magnesium hydroxide reacted with THPS.

**Figure 7:**
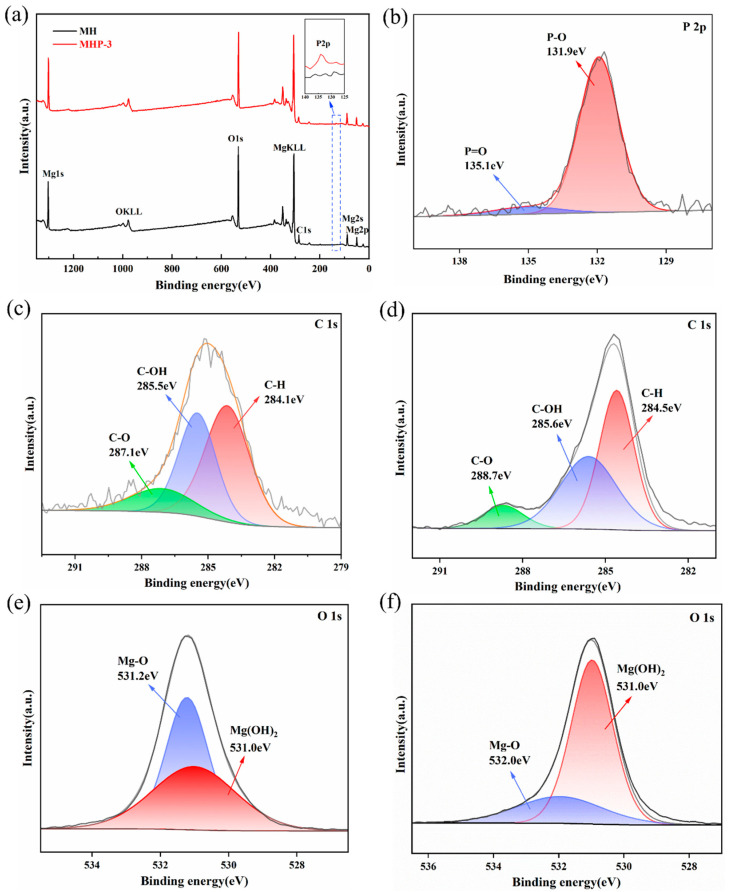
(**a**) XPS survey spectra; (**b**) P 2p XPS spectra of MHP-3; (**c**) C 1s XPS spectrum of MH; (**d**) C 1s XPS spectrum of MHP-3; (**e**) O 1s XPS spectrum of MH and (**f**) O 1s XPS spectrum of MHP-3.

In Figure 7, the XPS results of MH and MHP-3 are displayed. The XPS spectra of MHP-3 show signals corresponding to carbon, oxygen, phosphorus, and magnesium, and the new peak appears around 135 eV, corresponding to P 2p electron binding energy.

A typical characteristic of THPS is the formation of chemically stable P–C bonds [62]. High-resolution P 2p spectral analysis (Figure 7b) shows three distinct fitted peaks: a prominent peak centered at 131.9 eV, with smaller peaks at 135.1 eV, corresponding to the P atoms in the P–O and P=O bonds [63].

The C 1s spectrum of MH (Figure 7c) shows a peak at 287.1 eV, corresponding to C–O bonds. The bonding states of C ls (Figure 7d) at 288.7 eV correspond to the interaction of the C–OH groups in THPS with MH, resulting in the formation of C–O–Mg bonds. The bonding states at 285.6 and 284.5 eV are attributed to the C–OH and C–H bond structures inherent in THPS. With the addition of 3 wt.% THPS, the Mg–O bond structure shifted of the O 1s peak compared to pure MH, further indicating the chemical bond connection of THPS and MH. The modification reaction mechanism of THPS with MH is as follows:


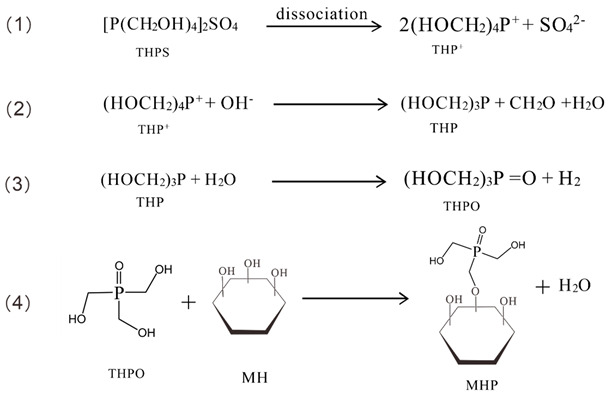


Firstly, THPS hydrolyzes to produce (HOCH_2_)_4_P^+^, and under alkaline conditions, the cation (HOCH_2_)_4_P^+^ decomposes into trimethylol phosphate (THP) and formaldehyde (CH_2_O). The THP is further oxidized by water to form trimethylol phosphate oxide (THPO) and H_2_. THPO subsequently forms a coordination complex with the solid surface of MH, resulting in a hydroxyl coordination product (MHP).

**Figure 8:**
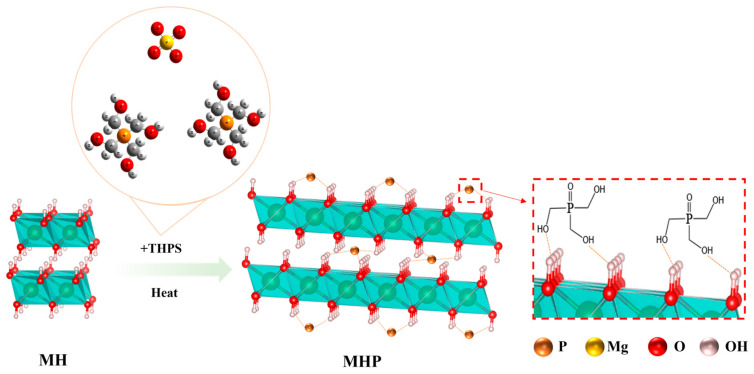
The diagrammatic sketch of the produced MHP.

After the modification, the diagrammatic sketch of the produced MHP is given in Figure 8.

### 3.3. Thermal Analysis of Modified MH

**Figure 9:**
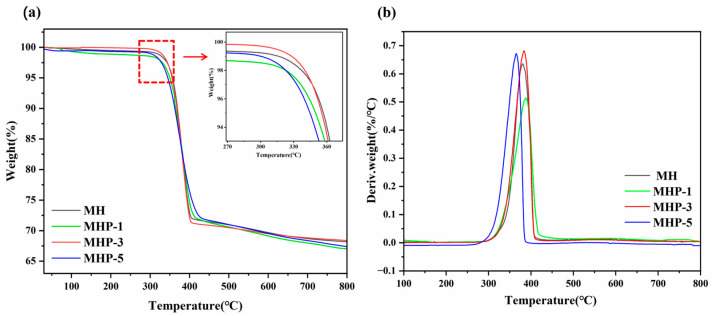
Thermogravimetric analysis of MH, MHP-1, MHP-3, and MHP-5 in N_2_. (**a**) TG and (**b**) DTG curves.

The samples’ high-temperature stability and charring rate were employed using thermogravimetric analysis (TGA). The thermal decomposition processes of MH, MHP-1, MHP-3, and MHP-5 from 30 to 800 °C in a nitrogen atmosphere are shown in Figure 9, with the relevant information organized and listed in Table 2. The temperature referred to a weight reduction of 5% (T_5%_) was used to represent the primary decomposition temperature, and T_max_ refers to its peak mass loss rate. The T_5%_ and the residual mass were derived from the TGA results, while T_max_ was determined from the corresponding DTG profiles. The major mass loss took place over the temperature range of 300–500 °C.

As for MH, the decomposition started at about 302.5 °C. The T_max_ is at 376.2 °C, with a total mass loss of approximately 31.8%. In contrast, MHP-3 showed an obviously improved thermal stability. The start of decomposition temperature increased to 325.0 °C, and the temperature at T_5%_ was 352.2 °C, which was higher than the MHP-1 (344.7 °C) and MHP-5 (345.4 °C), indicating that MHP-3 had better resistance to early-stage thermal degradation. It is due to phosphates generated from the THPS decomposition, forming a stable barrier on the MH surface that slows down thermal degradation. Meanwhile, the decomposition of MHP-3 produced H_2_O and other non-combustible gases, which can take away heat and dilute oxygen [64].

The T_max_ also shifted to a higher value (383.6 °C), suggesting enhanced resistance to thermal decomposition. Moreover, the total mass loss of MHP-3 was lower at 31.6%, compared to MHP-1 and MHP-5. The results show that the modification significantly improved the high-temperature stability of MH without compromising its high-temperature residue, which is beneficial for maintaining the protective barrier during combustion. The result suggests that the combination of THPS and MH can produce water vapor over a broad temperature range, benefiting the dissipation of combustion heat and dilution of flammable gases. Thus, the addition of P to MH improves both thermal stability and the degree of charring.

### 3.4. Flame-Retardant Properties of EVA Composites

#### 3.4.1. Fire Retardancy

LOI and UL-94 measurements are used to evaluate the flame retardancy [65]. Materials are regarded as non-combustible when the limiting oxygen index exceeds 26 [66]. The flame retardancy of the composites of LOI results is listed in Table 3. Both pure EVA and EVA/MH samples burned for more than 30 s, failing to obtain a UL-94 rating. By comparison, with the same amount of addition (with a 60 wt.% loading), the LOI values of EVA/MHP-1, EVA/MHP-3, and EVA/MHP-5 composites increased to 29.0%, 31.3%, and 30.5%, respectively, and reached a V-0 rating according to the UL-94 test. It is noted that the LOI value slightly decreases from 31.3% for EVA/MHP-3 to 30.5% for EVA/MHP-5. This may be attributed to excessive THPS loading, which could lead to particle aggregation, surface oversaturation, or disruption of the optimal MH–THPS balance, thereby slightly reducing the efficiency of char formation.

**Figure 10:**
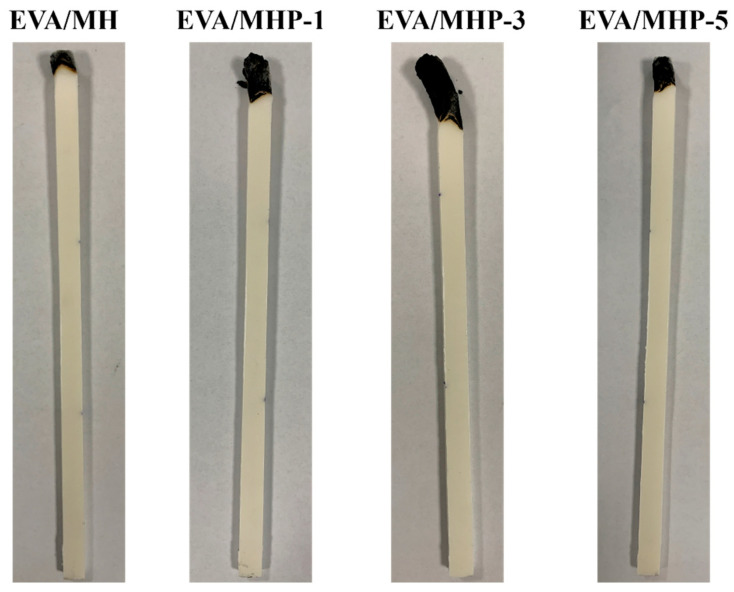
Digital images of neat EVA/MH and EVA/MHP composites after the LOI test.

Figure 10 shows pictures of pure EVA polymer and EVA/MH material after the LOI test. After burning, the formation of a carbon layer occurred on the end surface, isolating the oxygen from contact with the substrate. MH inclusion causes a rise in the LOI value of the EVA substrate, which results from the barrier effect preventing heat and volatiles. The EVA/MHP-3 material formed a tightly structured, uniform carbon layer with the most char produced, which led to enhanced heat dissipation and put out the fire, accordingly, showing the higher LOI values.

#### 3.4.2. Flame Retardancy Mechanism Analysis

**Figure 11:**
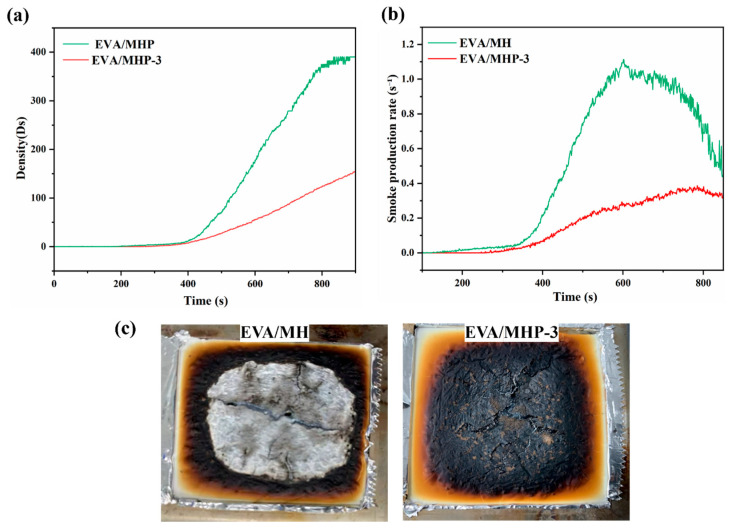
(**a**) Smoke density; (**b**) smoke production rate; (**c**) char residues of EVA/MH and EVA/MHP-3 composites.

A smoke density chamber was used to measure both the smoke density and the rate of smoke production of the composites, as shown in Figure 11. The EVA/MHP-3 composite exhibited a markedly lower maximum smoke density (Ds max = 155.4) compared to EVA/MH (Ds max = 411.3), corresponding to a reduction of approximately 62.2%. Furthermore, the smoke production rate of EVA/MHP-3 was substantially slower.

As observed in the char morphologies shown in Figure 11c, EVA/MHP-3 forms a dense, cohesive, and continuous char layer, in contrast to EVA/MH, which was nearly completely consumed. The char layer acts as an effective physical barrier, limiting the heat transfer, diffusion of oxygen, as well as release of combustible volatiles. The presence of the MgO in the residue promotes a bridging and sintering effect, further improving the structural integrity of the protective layer. In addition, the phosphonate groups introduced by THPS participate in charring reactions, catalyzing the production of a stable carbonaceous layer and increasing the carbon yield. In the gas phase, highly active radical species, such as H· and OH·, are scavenged by THPS-derived phosphorus-containing species (e.g., PO·, PO_2_·), thereby suppressing the chain–branching reactions responsible for sustaining combustion and smoke formation. Moreover, the release of incombustible gases such as H_2_O dilutes flammable gases in the pyrolysis zone, contributing to reduced smoke density and optical obscuration.

The coordinated action of radical quenching in the gas phase and char reinforcement in the condensed phase slows down the thermal degradation of the EVA matrix, slows down the release rate of decomposition products, while lowering the concentration of soot precursors in the flame region.

**Figure 12:**
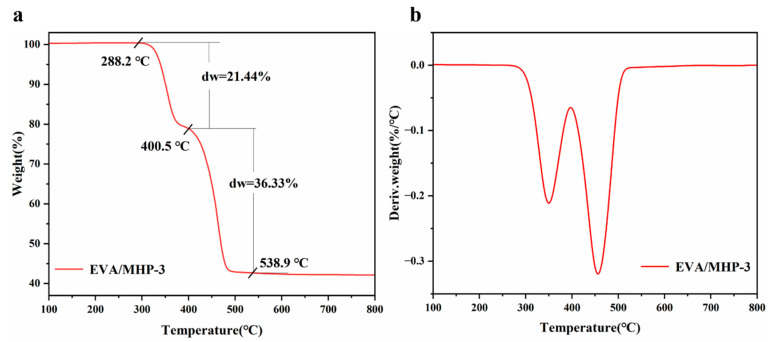
Thermogravimetric analysis of EVA/MHP-3 in N_2_. (**a**) TG and (**b**) DTG curves.

To achieve a deeper and more comprehensive insight into how EVA/MHP materials perform in terms of flame retardancy, the thermal degradation behavior was studied. The TGA and DTG curves of EVA/MHP-3 in N_2_ atmosphere are shown in Figure 12. The composite undergoes thermal decomposition predominantly in two distinct stages: 280–390 °C and 400–540 °C. The initial decomposition starts at 288.2 °C, with a weight loss of 21.44%. MHP decomposition produces phosphorus-containing compounds and releases water vapor, which dilutes flammable gases and interrupts radical chain reactions in the gas phase. The primary decomposition process of EVA/MHP-3 occurred mainly between 400 and 540 °C.

In this stage, the Mg(OH)_2_ degraded upon heating to form the MgO and water vapor, resulting in a weight loss of 36.33%, with a T_max_ of 455 °C. As the number of the MgO particles increases, a dense MgO-based char layer is covered by a layer, which functions as an effective physical barrier to suppress heat transfer as also oxygen diffusion, preventing further degradation and inhibiting dripping behavior in the condensed phase. Meanwhile, various radicals (e.g., OH·, H·) containing gas-phase radicals can interact with P free radicals generated by THPS decomposition, which suppresses gas-phase chain reactions and enhances the charcoal-forming effect. When the temperature approached 600 °C, the breakdown of the Mg(OH)_2_ was finalized.

**Figure 13:**
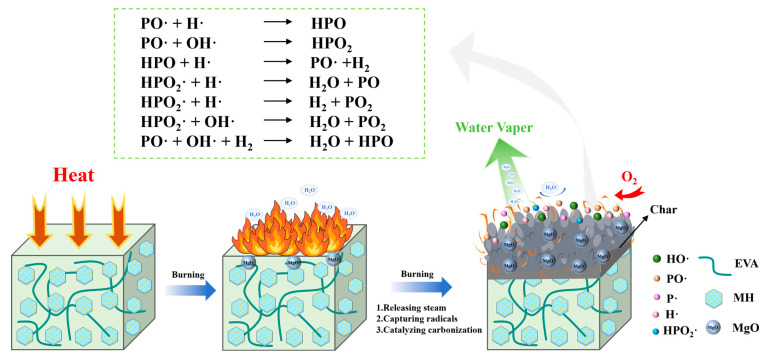
Flame-retardant mechanism diagram of MHP-3 in the combustion process.

The results show that MHP effectively improves the fire resistance of the composite materials, primarily via the synergistic effects of MH and THPS, indicating that the flame retardancy mechanism depends on both the MgO produced from thermal decomposition and the chain reaction of free radicals in the gas phase, preventing surface combustion of the polymer material. The mechanism responsible for flame-retardant behavior is schematically represented in Figure 13.

#### 3.4.3. Mechanical Performance of MHP

**Figure 14:**
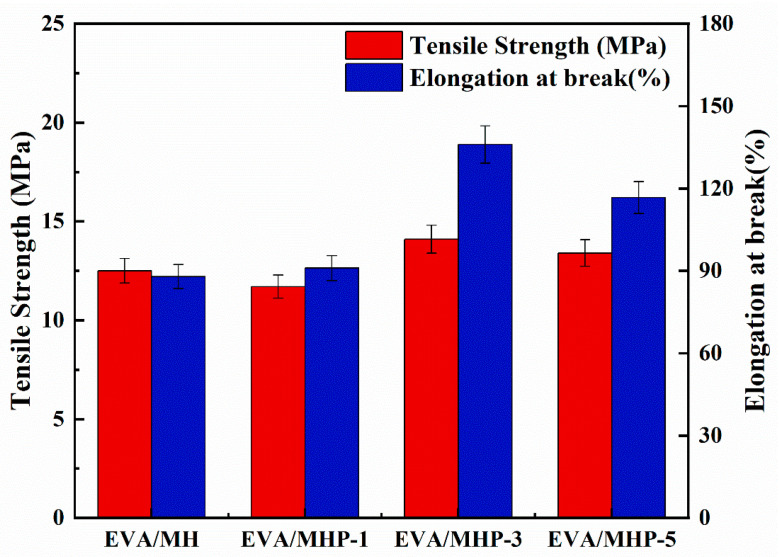
Tensile strength and elongation at break of EVA/MH, EVA/MHP-1, EVA/MHP-3, and EVA/MHP-5 composites.

A detailed assessment was conducted on the mechanical characteristics of EVA/MH composites, with tensile strength and elongation at break, as presented in Figure 14; relevant data are shown in Table 4.

Relative to the EVA/MH composite, the elongation at break of EVA/MHP-1, EVA/MHP-3, and EVA/MHP-5 all showed improvement. Among them, EVA/MHP-3 achieved the maximum break elongation of 136%, along with the maximum tensile strength, indicating that the addition of 3 wt.% THPS has a favorable influence on the composites’ mechanical properties. It is due to the even dispersion of modified MH in the EVA matrix, which helps avoid stress concentration and maintains the flexibility and strength of the composites. The results showed that the mechanical properties of the polymer were strengthened through the addition of modified MH, and the composites were suitable for materials used in wire and cable sheathing.

## 4. Conclusions

The hexagonal magnesium hydroxide nanoflakes with uniform morphology and excellent dispersion were prepared using a one-pot high-temperature hydrothermal method with THPS modification. Using XRD, FE-SEM, Raman spectra, XPS, and thermogravimetric analysis, the composition, morphology, and structure of the obtained nanoflakes were characterized.

Results indicated that the hexagonal THPS-modified MH nanoflakes had excellent flame-retardant performance. Meanwhile, the prepared EVA/MHP composites showed an increase in thermal stability and promoted char generation. Vertical burning and LOI test confirmed enhanced flame retardancy, with the LOI enhancing from 23.9% to 31.3% compared to pure MH. Digital photographs and thermogravimetric analysis of post-combustion residues were used to explain the potential flame-retardant mechanism. The residual char surface is composed of numerous spherically shaped the MgO particles, which form a compact protective layer for the underlying material during combustion. Additionally, water vapor and phosphorus-containing radicals released from EVA/MHP-3 during combustion effectively block the movement of heat, oxygen, and flammable species from the flame front to the underlying material. Overall, the incorporation of modified MH with superior dispersion resulted in considerable improvements in flame retardancy, reduced smoke production, and maintained the mechanical stability of the composites, thereby contributing to the enhancement of the application performance of eco-friendly magnesium hydroxide flame-retardants and offering insights into the further development of halogen-free flame-retardant systems.

## Figures and Tables

**Table 1:** Crystal data and grain size information from XRD patterns of MH, MHP-1, MHP-3, and MHP-5.

Samples	Temperature (°C)	I(001)/I(101)	Grain Size/nm	
			(001)	(101)
MH	60	0.71	17.04	21.92
MH	200	1.71	34.61	34.62
MHP-1	200	0.97	16.15	19.97
MHP-3	200	0.99	45.26	43.97
MHP-5	200	0.79	17.42	22.95

**Table 2:** T_5%_, T_max_, and residual weight at 800 °C of MH, MHP-1, MHP-3, and MHP-5.

Samples	T_5%_ (°C)	T_max_ (°C)	Residue at 800 °C (%)
MH	353.0	376.2	68.2
MHP-1	344.7	387.6	67.0
MHP-3	352.2	383.6	68.4
MHP-5	345.4	366.1	67.4

**Table 3:** LOI and vertical burning results of EVA, EVA/MH, and EVA/MHP composites.

Samples	Vertical Burning Test			
	t1/t2 (s)	Dripping/Ignition of Cotton	Rating	LOI (%)
EVA	>30	YES/YES	NR	19.8
EVA/MH	>30	NO/NO	NR	23.9
EVA/MHP-1	6.4/5.6	NO/NO	V-0	29.0
EVA/MHP-3	2.3/3.4	NO/NO	V-0	31.3
EVA/MHP-5	5.2/4.3	NO/NO	V-0	30.5

**Table 4:** Tensile strength and elongation at break of EVA/MH and EVA/MHP composites.

Samples	Tensile Strength (MPa)	StdDev (MPa)	Elongation at Break (%)	StdDev (%)
EVA/MH	12.5	0.2	87.9	4.8
EVA/MHP-1	11.7	0.3	91	9.7
EVA/MHP-3	14.1	0.4	136	0.4
EVA/MHP-5	13.4	0.4	116.7	15.7

StdDev: standard deviation.

## Data Availability

The original contributions presented in this study are included in the article. Further inquiries can be directed to the corresponding author.
